# 45 years German Society of Biological Psychiatry (DGBP)

**DOI:** 10.1007/s00702-023-02645-2

**Published:** 2023-05-04

**Authors:** P. Riederer, T. Kircher, G. Juckel, K. Domschke, A. Schneider, J. Deckert, P. Falkai

**Affiliations:** 1 Department of Psychiatry, Psychosomatics and Psychotherapy, Center of Mental Health, University Hospital Würzburg, Würzburg, Germany; 2 Department of Psychiatry, University of Southern Denmark, Odense, Denmark; 3 Department of Psychiatry and Psychotherapy, CMBB, University of Marburg, Marburg, Germany; 4 LWL-University Hospital of Psychiatry, Psychotherapy and Prevention Medicine at Ruhr University Bochum, Bochum, Germany; 5 Department of Psychiatry and Psychotherapy, Faculty of Medicine, Medical Center, University of Freiburg, Freiburg, Germany; 6 Department of Neurodegenerative Diseases and Geriatric Psychiatry, University Hospital Bonn, Bonn, Germany; 7 German Center for Neurodegenerative Diseases, Bonn, Germany; 8 Department of Psychiatry and Psychotherapy, LMU Munich, Munich, Germany; 9 Max-Planck-Institute of Psychiatry, Munich, Germany

**Keywords:** DGBP, German Society of Biological Psychiatry, WFSBP, DGPPN, AGNP, DFG

## Abstract

The foundation of a German Society of Biological Psychiatry (DGBP) was initiated at the Second World Congress of Biological Psychiatry of the WFSBP in Barcelona in 1978. Its mission was and is to promote interdisciplinary research on the biology of mental disorders and to translate results of biological research into clinical practice. During the presidency of Peter Falkai, its tasks were defined to improve the quality and support of biologically oriented research in Germany by the DFG (Deutsche Forschungsgemeinschaft; German Research Foundation), BMBF (Bundesministerium für Bildung und Forschung) and EU (European Union), to promote young researchers doing biologically oriented research, to improve on the diagnosis and therapy of mental disorders and to advise policy makers by taking part in legal processes. The DGBP has been a corporate member of the WFSBP from its beginning, became a cooperative member of the DGPPN (Deutsche Gesellschaft für Psychiatrie und Psychotherapie, Psychosomatik und Nervenheilkunde), later of the German Brain Council, and fostered relationships with other scientific societies. Over the past 45 years, more than twenty congresses were held in Germany and neighboring countries. Emerging from the pandemic, the DGBP is ready to continue its mission to promote interdisciplinary research on the biology of mental disorders with a focus on the development of young scientists and to translate results of biological research into clinical practice, with regard to pharmacotherapy in close cooperation with the Arbeitsgemeinschaft Neuropsychopharmakologie und Pharmakopsychiatrie (AGNP). In this sense, this article also aims to stimulate the cooperation of the society with other national and international partners and to foster new relationships with young scientists and professionals interested in the aims and goals of the DGBP.

## Founding of the DGBP

The 1970s were a time of psychiatry reform worldwide and also in Germany. In Germany, the German Bundestag had established a committee “Zur Lage der Psychiatrie in der Bundesrepublik Deutschland” (“On the state of psychiatry in the Federal Republic of Germany”), the recommendations of which were put into effect over the following years and contributed to seminal improvements in psychiatric care.

Part of these improvements were due to progress in the understanding of the biology and the pharmacological therapy of mental disorders. To reflect and further this development, international and national societies of biological psychiatry (World Federation of Societies of Biological Psychiatry [WFSBP], Deutsche Gesellschaft für Biologische Psychiatrie; German Society of Biological Psychiatry [DGBP]) and neuropsychopharmacology (Collegium Internationale Neuropsychopharmacologicum [CINP], European College of Neuropsychopharmacology [ECNP], Arbeitsgemeinschaft Neuropsychopharmakologie und Pharmakopsychiatrie [AGNP]) were founded.

The foundation of a German Society of Biological Psychiatry (DGBP) was initiated at the Second World Congress of Biological Psychiatry of the WFSBP in Barcelona in 1978.

German participants of the congress followed the call of the WFSBP General that delegates of national societies should take part in the WFSBP General Assembly. A declaration of intent to found a national society of biological psychiatry in Germany with a list of German participants was handed over to the WFSBP. This list consisted of the following persons: H. Beckmann, Bouchard, H. Erhardt, E.W. Fuenfgeld, H.J. Haase, A. Herz, R. Hopf, G. Huber, W. Keup, T.O. Kleine, J. Krieglstein, K.-J. Netter, F. Reimer, W. Seeler, E.H. Tremblau, W. Wesemann and P. Zimmermann.

On April 3rd, 1979, the founding assembly took place in Marburg, Germany, the members of which were H. Beckmann, H. Erhardt, E.W. Fuenfgeld, W. Keup, T.O. Kleine, J. Krieglstein, K.-J. Netter, F. Reimer, E.H. Tremblau, and P. Zimmermann.

Bylaws were established and a first executive board consisting of a president, a vice president, secretary and treasurer were nominated. The first regular meeting of the society was held in September 1979 in Weinsberg, Germany; at this time, the society consisted of 27 ordinary members.

## Mission of the DGBP

The focus of the DGBP over its 45 years essentially was twofold:

One was to promote interdisciplinary research on the biology of mental disorders with a focus on the development of young scientists.

The other was to translate results of biological research into clinical practice, with regard to pharmacotherapy in close cooperation with the Arbeitsgemeinschaft Neuropsychopharmakologie und Pharmakopsychiatrie (AGNP).

Its tasks, thus, were defined during the presidency of P. Falkai as follows:

1. Improvement of the quality and support of biologically oriented research in Germany by the DFG (Deutsche Forschungsgemeinschaft; German Research Foundation), BMBF (Bundesministerium fuer Bildung und Forschung) and EU (European Union),e.g., in the context of the DFG expert college of biological psychiatry
2. Promotion of young researchers doing biologically oriented research,e.g., by the organization of cutting-edge scientific congresses with scientific awards for young scientists
3. Improvement of knowledge for diagnosis and therapy of mental disorders,e.g., by participation in the development of national guidelines and organization of workshops in the context of the scientific congresses
4. Advising policy makers by taking part in legal processes,e.g., by commenting on legal drafts.

In its mission, the DGBP is supported by its publication organs, the European Archives of Psychiatry and Clinical Neuroscience and the Journal of Neural Transmission which encourage submissions by members and in particular junior scientists for special issues on topics of interest.

## Executive boards, sections and members of the DGBP

Over the years, representatives of 15 German university psychiatry departments acted as members of the executive board, thus documenting the strength of biologically oriented research in German psychiatry (Table 1).

**Table 1:** Boards of the DGBP since 1979

Time period	President	Vice President	Secretary	Treasurer
1979–1982	W. Keup (Berlin)	A. Hopf (Düsseldorf)	E. W. Fuenfgeld (Marburg)	H. Beckmann (Mannheim)
1983–1986	K. Heinrich (Düsseldorf)	A. Hopf (Düsseldorf)	E. W. Fuenfgeld (Marburg)	H. Beckmann (Mannheim)
1987–1990	H. Beckmann (Würzburg)	K. Heinrich (Düsseldorf)	G. Laux (Würzburg)	M. Ackenheil (Munich)
1991–1994	H. -J. Moeller (Bonn)	H. Beckmann (Würzburg)	G. Laux (Würzburg)	W. Gaebel (Düsseldorf)
1995–1998	P. Riederer (Würzburg)	H. -J. Moeller (Munich)	W. Gattaz (Mannheim) since 1996 J-C.. Krieg (Marburg)	W. Gaebel (Düsseldorf)
1999–2002	W. Gaebel (Düsseldorf)	P. Riederer (Würzburg)	J. -C-Krieg (Marburg)	N. Müller (Munich)
2003–2006	N. Müller (Munich)	W. Gaebel (Düsseldorf)	J. -C. Krieg (Marburg)	P. Falkai (Homburg/Saar)
2007–2010	P. Falkai (Göttingen)	N. Müller (Munich)	A. Heinz (Berlin)	S. Herpertz (Aachen)
2011–2014	A. Heinz (Berlin)	P. Falkai (Munich)	S. Herpertz (Rostock)	J. Deckert (Würzburg)
2015–2018	S. Herpertz (Heidelberg)	A. Heinz (Berlin) since 2016 F. Schneider (Aachen)	F. Schneider (Aachen) since 2016 T. Kircher (Marburg)	J. Deckert (Würzburg)
2019–2022	J. Deckert (Würzburg)	T. Kircher (Marburg)	K. Domschke (Freiburg)	G. Juckel (Bochum)
2023–2026	T. Kircher (Marburg)	G. Juckel (Bochum)	A. Schneider (Bonn)	J. Deckert (Würzburg)

Members represented 15 academic institutions throughout Germany

During the presidency of W. Gaebel, task forces were established which in the presidency of S. Herpertz were redefined as official sections with nominated speakers representing the spectrum of biological research of the society.

As of 2023, these sections (with speakers in brackets) are:

Imaging (Gruber/Heidelberg, Teipel/Rostock), Genetics and Endophenotypes (Gallinat/Hamburg, Schulz/Munich, Rujescu/Vienna), Neurophysiology (Pogarell/Munich, Hegerl/Frankfurt, Höppner/Rostock), Immunology (Endres/Freiburg, Steiner/Magdeburg), Biomarkers and Psychopharmacotherapy (Menke/Munich, Müller/Toronto, Himmerich/London), CSF Research (Bechter/Günzburg and Kurtcuoglu/Zürich), Experimental Psychiatry (Freund and Juckel/Bochum, Winter/Dresden), Molecular Dementia Research (Schneider/Bonn and Priller/Munich).

From the very beginning, members of the DGBP have been elected on an interdisciplinary basis. As such clinicians, biologists, chemists and psychologists have been accepted and are active as members. Members were not restricted to Germany, but also included members of other German-speaking countries over the years.

With the aim of promoting the development of new pharmacological treatments, there were institutionalized relationships with pharmaceutical companies as corporate members, sponsors of congresses and awards (Organon, Essex). During the presidency of S. Herpertz, institutionalized relationships with pharmaceutical companies have been terminated in accordance with a general tendency in psychiatry to avoid conflicts of interest. Currently, there are no corporate members anymore, and neither congresses nor awards are sponsored by pharmaceutical companies anymore. However, researchers working in pharmaceutical companies are welcome to become members and take part in the scientific congresses to promote scientific exchange between academia and industry.

Over the years, a number of outstanding German and international researchers in biological psychiatry has been awarded an honorary membership in the society.

They include in chronological order: E.-W. Fuenfgeld (1988), H.-J. Haase (1988), K. Heinrich (1988), H. Hippius (1996), W. Poeldinger (1996), A. Carlsson (1998), S. Hoyer (1998), P. Janssen (1998), J. Maj 1998, N. Matussek (1998), H. Beckmann (2000), E.-H. Tremblau (2000), H. Helmchen (2002), G. Huber (2002), P. Riederer (2007), W. Gaebel (2008), G. Gross (2008), B. Bogerts (2010), J.-C. Krieg (2010), J. Angst (2020).

## DGBP and WFSBP, the 2001 WFSBP Congress and the foundation “Nervenheilkunde“

The DGBP has been a corporate member of the WFSBP from its beginning. Members of the DGBP acted as president or vice president (H.-J. Moeller, P. Falkai) or treasurer (H. Beckmann, N. Müller) of the WFSBP. From 2004 to 2018, P. Riederer acted as chairman of the WFSBP-Task Force on “Biological Markers”. This group of experts organized and published several consensus reviews regarding biological markers in anxiety, depression, schizophrenia and dementia disorders during this period. In 2021, he was awarded the life time award by the WFSBP. Inside the WFSBP, the DGBP maintains close contact to German-speaking societies (Austrian, Swiss) and Western European societies (Belgium, France) having organized joint congresses with them (Table 2).

**Table 2:** DGBP congresses since 1979

Congress	City	Year
1. DGBP congress	Weinsberg	1979
2. DGBP congress	Düsseldorf	1982
3. DGBP congress and 1. Drei-Länder-Symposium	Lindau	1984
4. DGBP congress	Würzburg	1986
2. Drei-Länder-Symposium	Innsbruck	1988
5. DGBP congress	Berlin	1990
3. Drei-Länder-Symposium	Lausanne	1992
6. DGBP congress	Munich	1994
4. Drei-Länder-Symposium	Würzburg	1996
7. DGBP congress	Jena	1998
5. Drei-Länder-Symposium	Vienna	2000
8. DGBP congress	Düsseldorf	2002
1. West European congress	Bruges	2003
6. Drei-Länder-Symposium	Bern	2004
9. DGBP congress	Munich	2006
2. West European congress	Strassbourg	2007
7. Drei-Länder-Symposium	Göttingen	2008
3. West European congress	Berlin	2010
10. DGBP congress	Heidelberg	2012
11. DGBP congress	Aachen	2014
12. DGBP congress	Würzburg	2016
13. DGBP congress	Heidelberg	2018
1. Joint AGNP/DGBP congress	Berlin	2019
2. Joint AGNP/DGBP congress	Virtual online	2020
3. Joint AGNP/DGBP congress	Berlin	2022

During the presidency of H.-J. Moeller in the early 1990s and after careful preparation by H. Beckmann, the 7th WFSBP Congress took part in Berlin in 2001 with active participation of numerous members of the DGBP (with function in brackets) such as H.-J. Moeller (congress president), P. Riederer (chairman of the local organizing committee), W. Gaebel, P. Falkai, C. Krug, H. Grunze, M. Roesler (members of the local organizing committee).

The congress was formally organized by the “Verein zur Durchführung des 7th WFSBP Congress” (Association for the Organization of the 7th WFSBP Congress) P. Riederer as chair, H.-J. Moeller and W. Gaebel as vice chairs, J.-C. Krieg as secretary, N. Müller as treasurer and B. Ahrens and H. Grunze as advisors. It was a stunning success, scientifically and financially, allowing the establishment of the Foundation “Nervenheilkunde” in 2004 during the presidency of N. Müller with the aim of promoting young scientists in their neuroscience research.

The executive and supervisory board of the foundation “Nervenheilkunde” is nominated by the respective board of the DGBP. As of 2023, the executive board consists of J. Deckert and G. Juckel and the advisory board of A. Reif and K. Domschke. Because of the low interest rates over several years, the foundation on recommendation of the legal supervisor “Stiftungsaufsicht” has been transformed into a “Verbrauchsstiftung” allowing the use of the stock capital over a period of 10 years for the purposes of the foundation.

## DGBP and AGNP

Having been founded at about the same time and with overlapping agendas and members, the relationship between DGBP and AGNP was a matter of intense discussion from the very beginning.

On a suggestion of H. Beckmann, it was agreed during the presidency of H.-J. Möller that congresses should be held alternatively in early October with the DGBP holding its congresses usually at the university of its president and the AGNP first at Erlangen and later at Munich. During the presidency of P. Falkai in the late 2000s, merging of the DGBP with the AGNP was proposed. However, this was not supported by the general assembly due to the different focus of the missions (DGBP: biology of mental disorders, AGNP: Pharmacotherapy of mental disorders).

Finally in 2016, the boards of the two societies (from the DGBP S. Herpertz, F. Schneider, T. Kircher and J. Deckert) agreed to hold joint annual congresses at Berlin as workshop congresses for young scientists halfway between the annual, more clinically oriented congresses of the Deutsche Gesellschaft für Psychiatrie und Psychotherapy, Psychosomatik und Nervenheilkunde (DGPPN).

The first such DGBP/AGNP congress was organized by the AGNP at Berlin, in March 2019. The concept including method workshops and junior scientist symposia was well accepted. However, due to the COVID pandemic, the rhythm of the congresses over the following years was irregular with a first virtual congress organized by the DGBP in 2020 and the next live congress organized by the AGNP not until 2022.

## DGBP and DGPPN, AWMF, GBC

The DGBP is a cooperative member of the DGPPN. As such, it actively contributes to the annual congresses and closely cooperates on guidelines and policy statements.

The DGBP is a member society of the German “Arbeitsgemeinschaft der Wissenschaftlichen Medizinischen Fachgesellschaften” (AWMF) and as such is sending delegates to national S3 guideline committees, e.g., on schizophrenia, depression, suicidality, addiction, anxiety disorders and ADHD.

Recently, it has become a member of the German Brain Council (GBC) the mission of which is to promote brain research with the aim to improve diagnosis and therapy of brain disorders. Members such as P. Falkai (president) J. Deckert and A. Reif have contributed to the mission statement for policy makers and the public at large.

## DGBP and DFG

The Deutsche Forschungsgemeinschaft (DFG) as the central peer-review funding agency of basic and translational research is based on an expert college structure supervising the review process and recommending funding of individual grant applications.

A major achievement of the DGBP has been to convince the DFG to establish an expert college on Biological Psychiatry. Current members (2023) of this expert college are A. Fallgatter, K. Domschke and S. Riedel-Heller. Proposals of the candidates over the years were made in close contact with the DGPPN but also other societies with similar missions such as the Society of Neuroscience.

The existence of this expert college over the years has allowed researchers in Biological Psychiatry to submit their research proposals to expert peers and, thus, has contributed to the increase of excellent research on the biology of mental disorders in Germany.

Thus, members of the society were able to establish DFG-funded research networks such as collaborative research centers, e.g., the CRCs anxiety (TRR 58) and addiction (TRR 265), research groups (RG), e.g., the RGs ADHD (CRG 125)), Genotyp-Phenotyp Correlations (CRG 241), Affective Disorders (RG 2107) and research training groups, e.g., the RTG Emotions and Approach/Avoidance (RTG 1253), but also BMBF funded research networks and to compete successfully for EU grants of the various Horizon and EraNet programs.

## DGBP-Congresses, Congresses with German-speaking countries (Drei-Länder-Symposia), Congresses with Western European Societies and Congresses with the AGNP

A central element of the DGBP’s work was the biannual congress allowing scientific exchange at a personal level and providing a forum for young scientist to meet their peers and role models and some of them obtaining one of the prestigious junior scientist awards.

Over the past 45 years, more than 20 congresses were held in Germany and neighboring countries (Table 2).

Congresses were held biannually, some of them as joint congresses with other European societies (Austrian, Swiss, Belgium, French) and most recently together with the AGNP at Berlin.

During the COVID pandemic and as a compensation for the lack of the annual congress, a virtual online seminar series was offered by representatives of the DGBP sections. As a consequence of the COVID pandemic, bylaws were complemented in 2022 to allow for virtual meetings and congresses.

A particularly emotional moment was during the 2016 congress at Würzburg when the president S. Herpertz for the society extended her sympathy and apology to the descendants of Margarete Höppel, a victim of the so-called “euthanasia” program during which more than 200,000 German citizens with a mental disorder were killed between 1940 and 1945, on occasion of the renaming of the address of the Department of Psychiatry in Würzburg to Margarete-Höppel-Platz 1.

## Promotion of young scientists

To promote young scientists in their clinical and basic research, a number of different attractive honors were awarded. These include research and junior scientist awards, poster prizes and stipends for the participation in congresses and workshops. The award winners came from the complete spectrum of our members and included physicians, biologists, chemists and psychologists. Many of the award winners of the research and junior scientist awards later on became established scientists, professors and chairs and contributed to our knowledge of the biology of mental disorders (Table 3).

**Table 3:** Over the past 35 years, many, mostly young junior scientists obtained one of the DGBP research or junior scientist awards

Year	Award	Winner	University
1988	Organon	D. Riemann	Konstanz
1990	Organon	J. Kornhuber B. Bondi/B. Ertl U. v. Bardeleben/E. Klieser	Würzburg Munich Freiburg, Düsseldorf
1992	Organon	H. Beckmann, S. Cohen	Würzburg, Düsseldorf
1993	Organon	H-J. Haug B. Ahrens, R-D. Stieglitz	Basel Freiburg
1994	Organon	W. Mueller	Frankfurt
1996	Organon	C. Haass, J. Bauer	Munich, Freiburg
1998	Organon	J. Deckert, C. Behl	Würzburg, Munich
2000	Organon	J. Wiltfang	Göttingen
2002	Organon	G. Stöber, B. Lutz	Würzburg, Munich
2006	Organon	T. Frodl, T. Kircher	Munich, Aachen
2008	Essex	A. Reif, O. Gruber, C. Otte	Würzburg, Göttingen, Berlin
2014	Junior Scientist	K. Domschke,M. Walter,A. Hasan	Würzburg, Magdeburg, Munich
2016	Junior Scientist	B. Straube,L. Schilbach	Marburg, Munich
2018	Junior Scientist	D. Bzdok, D. Endres	Aachen, Freiburg
2019	Junior Scientist	S. Kittel-Schneider, D. Hirjak	Würzburg, Mannheim
2020	Junior Scientist	R. Redlich, B. Besteher	Halle, Jena
2022	Junior Scientist	P. Bach, N. Freund, G. Gryglewski, M. Schiele	Mannheim, Bochum, Wien, Freiburg

Many of them went on to become established scientists contributing to our understanding of the biology of mental disorders.

## DGBP and mental health

Emerging from the pandemic, the DGBP is ready to continue its mission to promote interdisciplinary research on the biology of mental disorders with a focus on the development of young scientists and to translate results of biological research into clinical practice, with regard to pharmacotherapy in close cooperation with the Arbeitsgemeinschaft Neuropsychopharmakologie und Pharmakopsychiatrie (AGNP).

It will do so by making extensive use of the competence of its members organized in sections based on state-of-the-art methods of the molecular (genetics, immunology) and systemic neurosciences (imaging and neurophysiology), but also clinical sciences (psychotherapy, pharmacotherapy, stimulation therapies). A junior scientist section will be established to give junior scientists a stronger voice.

It will do so in close concert with other societies such as the WFSBP with P. Falkai as vice president and president-elect and other national Biological Psychiatry societies, the DGPPN and the AGNP, but also the GBC and the Society of Neuroscience. International cooperation in a globalized world will be essential.

Promotion of young scientists will become more and more a central focus in a more and more competitive environment and competition with other disciplines for the brightest trainees. Congresses but also virtual training programs like the DGBP lecture series and stipends and award will be tools also in the future.

This will be done in close cooperation with the DFG and other funding agencies and their efforts to develop young scientists, e.g., by structured training programs.

More than in the past, the DGBP will also aim at translating scientific into clinical progress, e.g., by participation in clinical guidelines. It, thus, will support the DGPPN initiative for a 2nd guideline on “Apparative and laboratory diagnosis in Psychiatry”. Members of it contribute to the currently established German Center of Mental Health (DZPG).

The DGBP and its members are ready to contribute to the translation of the highly dynamic scientific progress in our knowledge on the molecular and systemic biology of mental disorders into clinical practice to the benefit of patients with mental disorders.

In this sense, the authors aim to stimulate the cooperation of the society with other national and international partners and to foster new relationships with young scientists and professionals interested in the aims and goals of the DGBP.

## Data Availability

There was no data generated to write this historical article. The data included in this article is taken from the archives of the scientific societies and accesible to everyone.

